# Spatial spillover analysis of a cluster-randomized trial against dengue vectors in Trujillo, Venezuela

**DOI:** 10.1371/journal.pntd.0008576

**Published:** 2020-09-03

**Authors:** Neal Alexander, Audrey Lenhart, Karim Anaya-Izquierdo

**Affiliations:** 1 MRC Tropical Epidemiology Group, Department of Infectious Disease Epidemiology, London School of Hygiene and Tropical Medicine, London, United Kingdom; 2 Vector Biology Department, Liverpool School of Tropical Medicine, Liverpool, United Kingdom; 3 Department of Mathematical Sciences, University of Bath, Bath, United Kingdom; Fundacao Oswaldo Cruz, BRAZIL

## Abstract

**Background:**

The ability of cluster-randomized trials to capture mass or indirect effects is one reason for their increasing use to test interventions against vector-borne diseases such as malaria and dengue. For the same reason, however, the independence of clusters may be compromised if the distances between clusters is too small to ensure independence. In other words they may be subject to spillover effects.

**Methods:**

We distinguish two types of spatial spillover effect: between-cluster dependence in outcomes, or spillover dependence; and modification of the intervention effect according to distance to the intervention arm, or spillover indirect effect. We estimate these effects in trial of insecticide-treated materials against the dengue mosquito vector, *Aedes aegypti*, in Venezuela, the endpoint being the Breteau index. We use a novel random effects Poisson spatial regression model. Spillover dependence is incorporated via an orthogonalized intrinsic conditional autoregression (ICAR) model. Spillover indirect effects are incorporated via the number of locations within a certain radius, set at 200m, that are in the intervention arm.

**Results:**

From the model with ICAR spatial dependence, and the degree of surroundedness, the intervention effect is estimated as 0.74—favouring the intervention—with a 95% credible interval of 0.34 to 1.69. The point estimates are stronger with increasing surroundedness within intervention locations.

**Conclusion:**

In this trial there is some evidence of a spillover indirect effect of the intervention, with the Breteau index tending to be lower in locations which are more surrounded by locations in the intervention arm.

## Author summary

Control methods for dengue, and other diseases which are transmitted by mosquitoes, are often tested in cluster-randomized trials. This means that whole groups of people, often defined by geographical area, are randomly allocated to receive the control method or not. These control methods often have a mass effect so that they may be stronger if applied to a whole area together. However, if the areas (clusters) are not very far apart then the effects may ‘spill over’ from one to another. In this paper we use a new spatial statistical method to re-analyse data from a cluster-randomized trial which was done in a town in Venezuela. The idea was to use insecticide treated curtains and water tank covers to try to control the mosquitoes which transmit dengue. To assess the spillover effect we calculate how much each location was surrounded by locations which got the control method. We found some evidence that the greater the surroundedness then the stronger the effect of the intervention.

## Introduction

Cluster-randomized trials have been increasingly used for interventions against infectious—and particularly vector-borne—diseases because of their ability to capture mass or indirect effects [1]. For example, people who live close to bednet-users may have lower average malaria incidence, even if they do not use a bednet themselves [2–4]. Standard methods for analysis of cluster-randomized trials assume that clusters are independent, so that any mass effects pertain only within each cluster. Such independence may be difficult to achieve, however, given that diseases may be spatially correlated over kilometres [5], or even tens of kilometres [6]. In fact, the scale of spatial correlation may be unknown when designing the trial. It may therefore be useful to adjust for any between-cluster effects when comparing the arms of the trial. This is to see such ‘spillover’ effects as a problem, or statistical nuisance parameters. On the other hand, the ability to measure such effects could be an opportunity, because their existence would be a positive feature of the intervention.

Various methods have been used for spatial analysis of cluster-randomized trials but they have generally been ad hoc additions to a standard analysis. We have developed a method which simultaneously incorporates both the cluster-randomization design, and the spatial configuration of the individuals and clusters. We distinguish two types of spatial spillover effect that may occur in a cluster-randomized trial.

The first is between-cluster dependence in outcomes, which we call *spillover dependence*. This means spatial autocorrelation across different clusters. In turn, spatial autocorrelation is a way to quantify Tobler’s first law of geography that “everything is related to everything else, but near things are more related than distant things” [7]. For example, in a cluster-randomized trial of a typhoid vaccine, individual risk of typhoid was associated with the risks of people living nearby [8]. The second type of spillover effect is an increase or decrease of the intervention effect, depending on the proximity of individuals in the control arm to those in the intervention arm: we call this *spillover indirect effect*. For example, in a post hoc analysis of a trial of bednets in Haiti, the proportion of containers positive for *Aedes* mosquitoes was found to be lower in those control arm houses which were within 50m of a house in the bednet arm [9]. In the current paper we estimate these effects in a previously reported cluster-randomized trial of insecticide-treated materials against dengue mosquito vectors in Venezuela [10].

## Materials and methods

### Trial design and interventions

The trial was carried out between January and November 2003 in the Santa Rosa suburb of Trujillo town, Trujillo State, Venezuela [10]. All households were eligible to participate, and 99.5% (1116/1122) agreed to do so. Of these, 1091 have spatial coordinates and are included in the current analysis. Eighteen geographically defined clusters of households were randomized to insecticide treated materials or to control. This sample size was chosen on the basis of the objectives of the original trial. The current analysis uses all available data without a further power calculation. The interventions were applied at house level. Intervention houses received i) PermaNet curtains, with netting treated with long lasting insecticide, specifically, deltamethrin at 50 mg/m^2^ (Vestergaard-Frandsen, Denmark), and ii) circular water jar covers made of the same PermaNet netting with an elastic rim. Curtains were hung loosely at the windows. Covers were provided for all household water drums (typically 150-200 l), where most vector breeding occurred. The curtains were impregnated again after five to six months with lambacyhalothrin. Control houses received no interventions, and therefore the study was not blind.

The clusters were pair-matched taking into account housing conditions—such as density, typical house size, and the condition and number of walls—and baseline values of Breteau index and house index. The Breteau index is the number of water containers per 100 houses that are positive for immature stages—larvae or pupae—of the mosquito vector species, *Aedes aegypti*. The house index is the percentage of houses with any immature stages. This matching was semi-quantitative, with baseline Breteau index being the most important variable, due to the infeasibility of simultaneously matching all pairs closely on all variables. This approach lacked reproducibility but, at the time, constrained randomization was not a well-established option for cluster-randomized trials [11]. The primary outcome was the Breteau index.

### Georeferencing and spatial data processing

**Fig 1:**
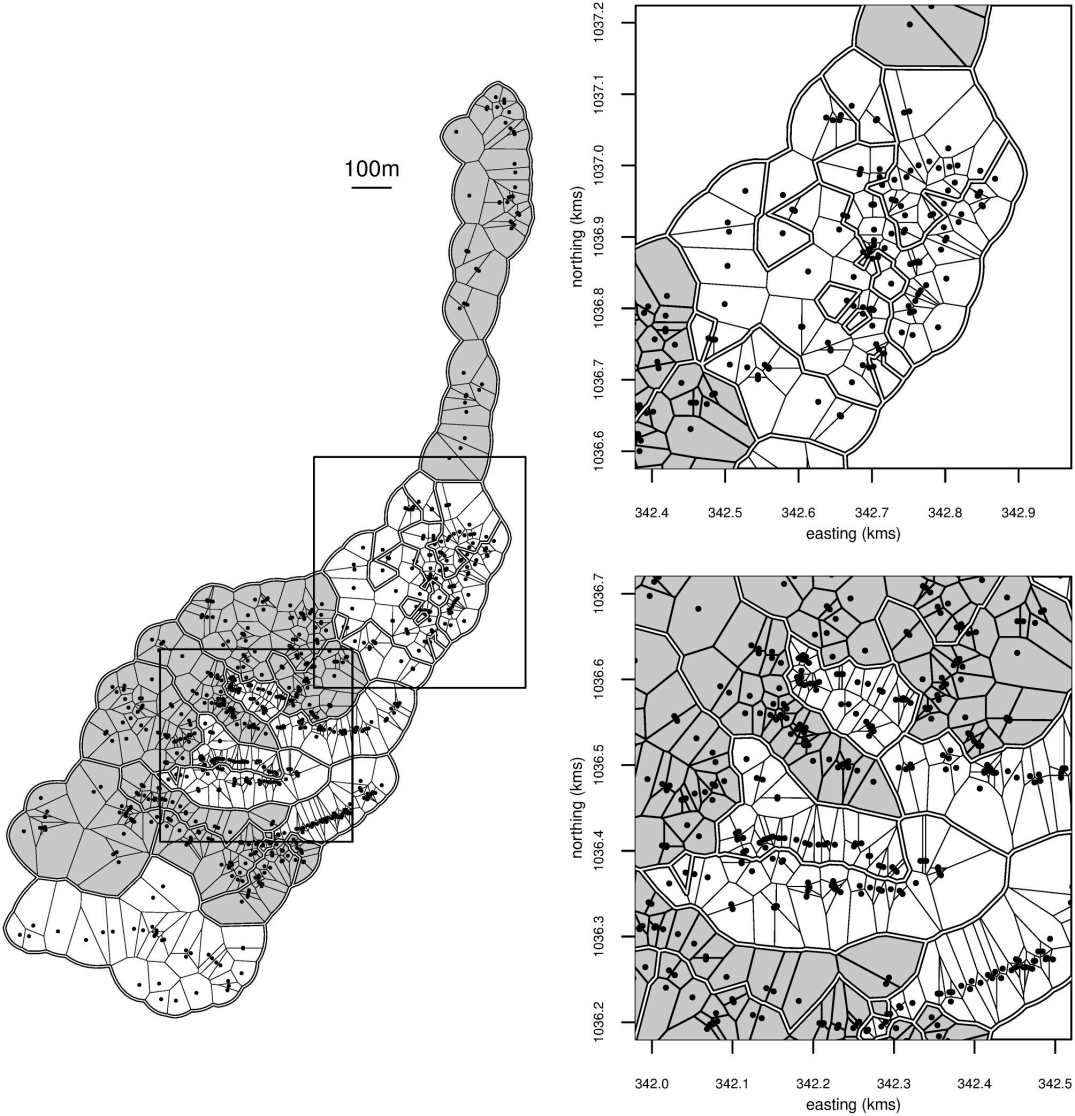
Spatial distribution of individuals in the Trujillo trial where each point represents a location, i.e. a house or, in the case of identical coordinates, a set of houses. There is one tile for each point, obtained using by Dirichlet tessellation. Dark tiles are intervention locations, white ones are control. Double lines are tile borders between clusters. The insets to the right are zoomed versions of the areas inside the rectangles.

**Fig 2:**
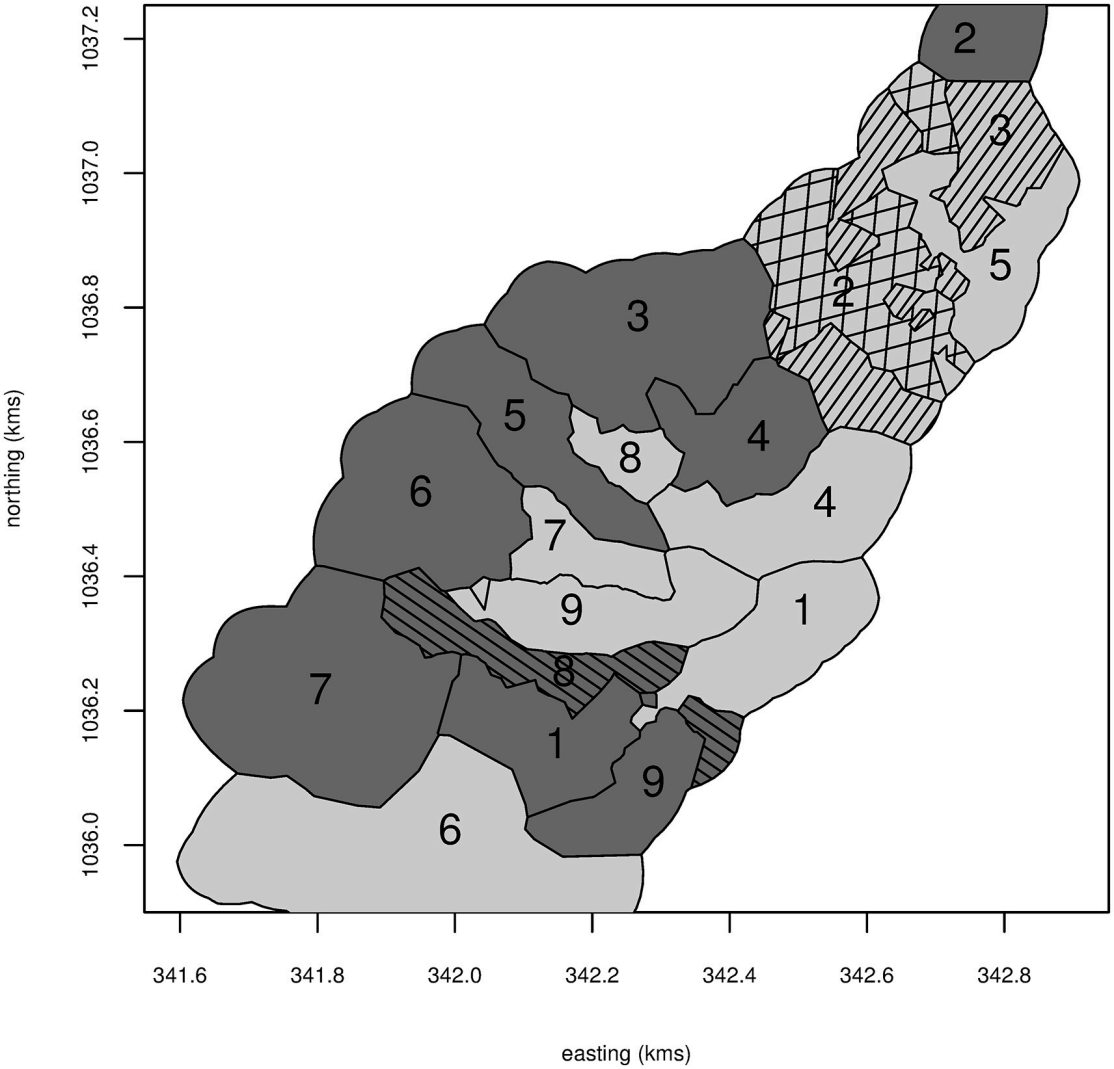
Pairing of clusters in the Trujillo trial. Darker clusters are in the intervention arm. Clusters with the same number are in the same matched pair (one in the intervention and one in the control arm). Hatching is used to show different sections of clusters which appear to be non-contiguous, due to limited GPS accuracy and steep terrain.

**Fig 3:**
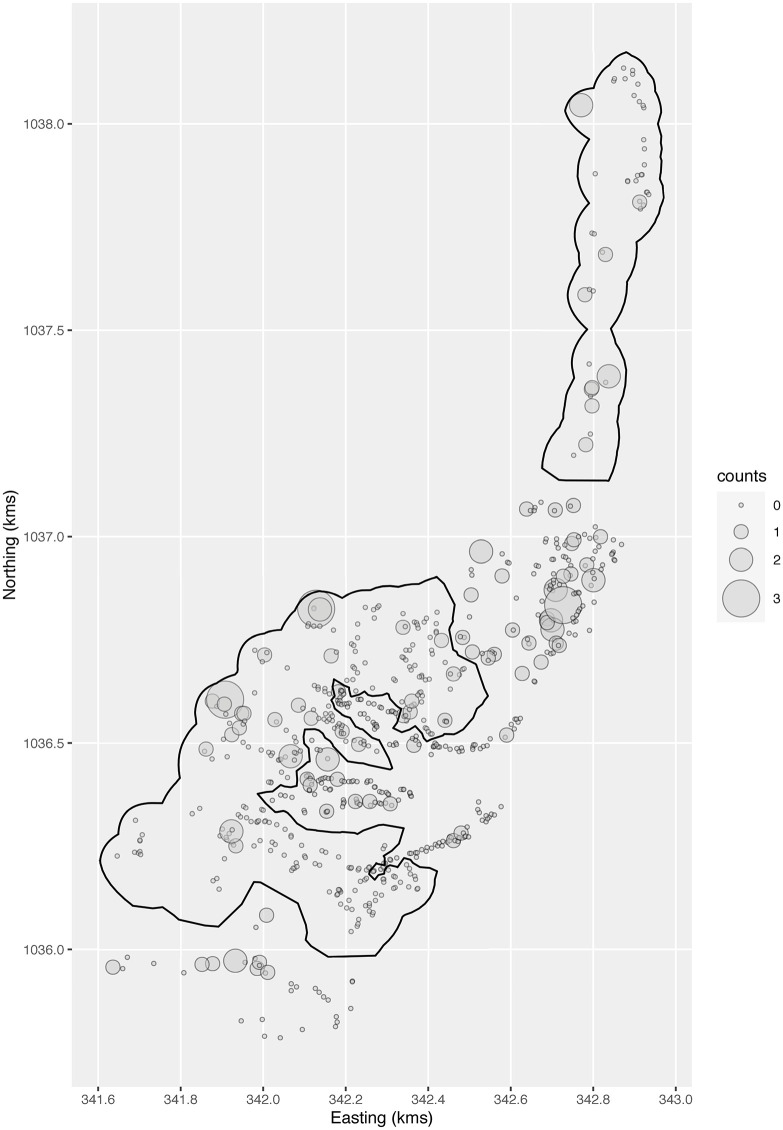
Number of containers positive for immature stages of the mosquito vector species *Aedes aegypti*, at each location, plotted at the centre of the corresponding tile. The Breteau Index, the endpoint of the trial, is the number of such containers per 100 houses. The dark solid lines surround the intervention clusters.

**Fig 4:**
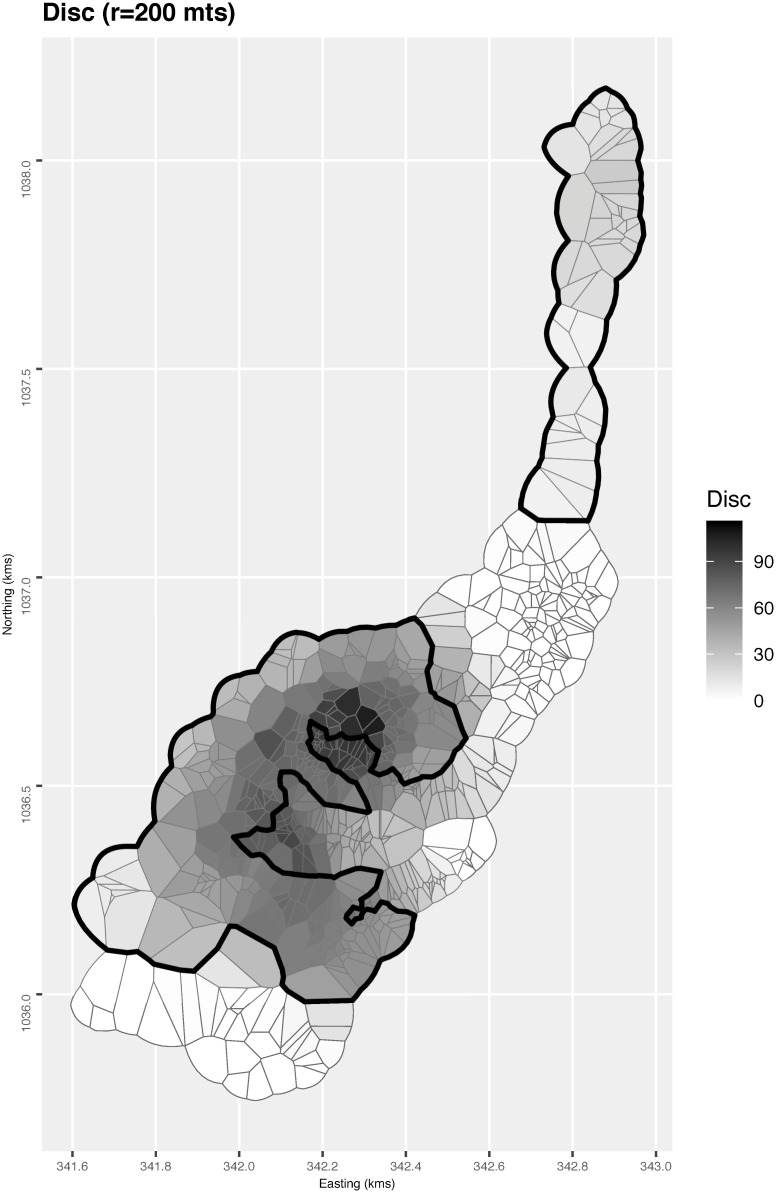
Choropleth map of the Dirichlet tiles around each location. Darker shadings indicate greater surroundedness by intervention locations according to the disc measure, i.e. number of intervention locations within a radius of 200m. Control regions are those inside the white boundary.

Residences were geo-referenced in terms of latitude and longitude using a hand-held Global Positioning System (GPS). For analysis the coordinates were converted to Universal Transverse Mercator (UTM) zone 19, with the World Geodetic System 1984 (WGS84) datum. Of the 1116 consenting households, 1091 were georeferenced. At the final survey, 730 had non-missing values for the number of water containers positive for immature vector stages. Households with identical coordinates were merged, giving 702 distinct locations. To define the neigbourhood structure we use the so-called Dirichlet tessellation [12] which partitions the area into a set of convex polygons called tiles, each being the subset of the area for which the given location is closer than any other. A set of points enclosing the study region is used to externally truncate the tiles [13]. Figs 1–4 were generated by the authors from said GPS data and processing.

### Statistical methods

The statistical methods for the spatial analysis are described in detail in S1 Text. In brief, we use spatial regression models with Gaussian random effects, where the individual outcomes have marginal distributions overdispersed relative to the Poisson distribution. The random effects represent spillover dependence in terms of an intrinsic conditional autoregression (ICAR) model [13], modified via an orthogonalization which i) avoids spatial confounding [14] and ii) ensures that the relevant model parameter can be interpreted as a between-arm ratio of expected values. Model equations are summarized in S1 Text. Our models are constructed to have the ‘constant variance property’, so that intervention effects are estimated efficiently [15].

Inferences are made from Bayesian methodology for generalized linear mixed models (GLMMs) [16] and integrated nested Laplace approximations (INLA) [17]. To compare goodness of fit of different models, we use the logarithm of the pseudo-marginal likelihood (LPML), calculated from the data’s conditional predictive ordinates [18]. Prior distributions of the parameters, and further information on goodness of fit measures, are included in S1 Text. We define two locations to be neighbours if and only if their corresponding Dirichlet tiles share a line boundary or even a point (so called queen type neighbours). In the Bayesian framework, the prior distributions are updated based on the model and data, to yield posterior distributions, from which point and interval estimates (credible intervals) are obtained.

A spillover indirect effect is an impact on an index location by surrounding locations in the intervention arm. We measure surroundedness in terms of the number of locations, within a certain radius of the index location, that are in the intervention arm. We call this the ‘disc’ measure of surroundedness. A spillover indirect effect may occur whether the index location is in the control arm or the intervention arm, and here we allow the magnitude of these effects to differ. The exponential of each regression coefficient of the disc measure is interpreted as the factor by which the expected Breteau index changes for each additional surrounding location. We show the results in terms of the ‘total intervention effect’ and the ‘pairwise intervention effect’ [15]. Both of these measures incorporate the direct effect of the intervention, and its spillover indirect effects mediated by surroundedness. The total intervention effect is the between-arm ratio in the sum of expected rates. The pairwise intervention effect is the between-arm ratio of expected rates for two locations with the same specified value of surroundedness, in opposing arms. For the current trial, they are between-arm ratios of expected Breteau index. A protective effect corresponds to a value less than 1.

### Ethics

Ethical approval for the original trial was given by the Universidad de los Andes, Venezuela, and the Liverpool School of Tropical Medicine, both in 2003. The former does not have a reference number, while for the latter it is 03.27.

## Results

The study area of Trujillo town is shown in Fig 1, and the cluster pairings in Fig 2. The randomized allocation has agglomerated the clusters into two contiguous regions per arm, with one of the control regions being interdigitated with one of the intervention regions. Fig 3 shows the number of positive containers at each location. Crudely, the Breteau index over all control locations is 16.2 (60 positive containers in 370 houses at 360 locations), and 12.8 over all intervention ones (46 positive containers in 360 houses at 342 locations), giving a ratio, or effect measure, of 0.79. From the model with ICAR spatial dependence and the disc measure of surroundedness, the intervention effect is 0.74, with a 95% credible interval of 0.34 to 1.69.

In terms of possible spillover indirect effect, crudely, the Breteau Index is 10.8 in those control locations with at least one intervention neighbour, and 18.3, or 1.7 times higher, in those control locations with no intervention neighbours. Comparing the latter subgroup with the intervention locations gives a stronger effect measure of 0.70 (12.8/18.3). Fig 4 shows a less crude measure of surroundedness, the number of intervention locations within a 200m radius. High values of this disc measure are concentrated in the central part of the study area. In other words, the locations in that central part (in both arms) are largely surrounded by intervention ones. Again, this suggests a possible influence on the intervention effect.

**Fig 5:**
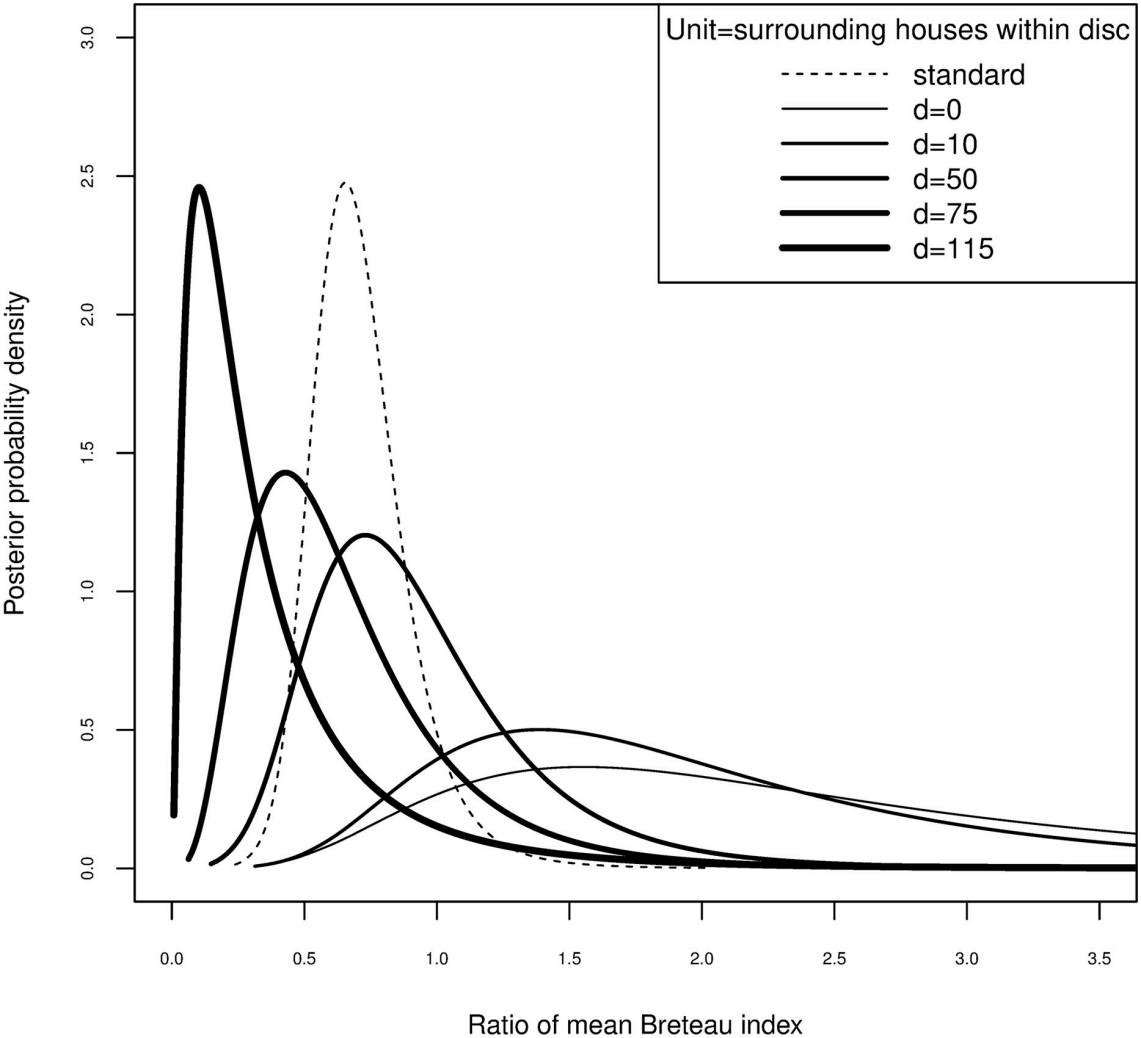
Pairwise intervention effect by surroundedness. Posterior distributions of the pairwise intervention effect (solid thick lines) for increasing values of surroundedness within intervention locations. The thickness of the solid lines is proportional to this surroundedness. The posterior distribution of the intervention effect from the standard (non-spatial) model is plotted with a dotted line.

The pairwise intervention effect was estimated by allowing the intervention effect to vary with the surroundedness by intervention locations. Fig 5 shows the posterior distributions of the pairwise intervention effect for various values of the disc measure of surroundedness. For this model, using a radius of 200m, the LPML is -0.419. Models with radius 100m or 300m had similar but slightly lower (less well-fitting) values of -0.421 and -0.422, respectively. As surroundedness increases, the posterior of the pairwise intervention effect shifts to the left and away from one, giving more evidence of an intervention effect in the corresponding subgroups.

## Discussion

Insecticide-treated materials (ITMs) may exert mass effects against mosquitoes. For *Aedes*, this has been found in several [19–21], but not all [22, 23], of the published cluster-randomized trials. Spillover indirect effect is a specific kind of mass effect, in which the intervention influences nearby areas which did not necessarily receive it. Some evidence for this kind of effect has previously been found in a trial in Haiti [9], as well as the one in Venezuela [10] which is the subject of the current paper. Trials of ITMs against *Anopheles* vectors of malaria have found mass effects [24–28]. Evidence of a more specific spillover indirect effect of insecticide-treated materials has also been found for *Anopheles* vectors of malaria, in a cluster-randomised trial of bed nets [29]. In houses within 300m of an intervention village, the abundance of *Anopheles funestus* was less than half that in houses which were 600m or more away. This was a secondary analysis of a trial in which the main endpoints were child mortality and morbidity [30], and did not include cluster as a factor, nor take into account spatial autocorrelation. This analysis found a smaller effect on *Anopheles gambiae*, consistent with the findings of Sinka et al. [31].

In the current paper we used novel methods to re-analyse data from a trial of insecticide-treated materials against the dengue vector *Aedes aegypti* [10], taking into account spatial structure as well as the cluster-randomized design. We found some indication of an indirect intervention effect, in the form of an inverse association between infestation in control houses and the degree to which they were surrounded by houses in the intervention arm, as opposed to the control arm. Evidence for an intervention effect is stronger in those houses which are most surrounded by intervention ones, and progressively weaker in those which are less surrounded (Fig 5). This may well be because clusters abutted each other, usually separated by no more than the width of a street. Defining clusters so close together may hinder the estimation of environmental risk factors which vary over a larger scale, although this would be secondary to estimating the intervention effect.

A related concern is that of geographical balance. If spillover indirect effects are a concern at the design stage, then it may be beneficial to limit the contiguity of clusters in the same arm, e.g. by constraining the randomization [11, 32]. However, the notion of geographical balance may be hard to quantify. For example, despite lying in just two contiguous regions per arm, 17 of the 18 Trujillo clusters border at least one in the opposite arm. One approach may be to impose a minimum proportion of between-cluster boundaries which separate clusters in different arms.

The statistical method is currently limited to data which are georeferenced as points, as opposed to areas, and which are counts, as opposed to being binary or continuous. Also, locations must currently be specified in two dimensions, rather than three. Depending on the application, it could be relevant to use distance in three dimensions (including elevation). If so, then the measures of surroundedness would need to be generalized, for example the disc measure would become spherical. In terms of software implementation, code for the R software has been provided with the companion paper [15], but this requires several detailed components to be specified, and has not yet been developed as an R package.

A recent systematic review [33] concluded that ‘there is no consensus on how to account for spatial effects with in CRTs [cluster-randomized trials] and more work needs to be done to evaluate and develop spatial methodology within the context of a range of CRTs.’ The original analysis of the Trujillo trial [10] included a secondary post hoc spatial analysis of baseline-infested houses in the control arm which became negative, and found that those within within 50m of a house in the intervention arm were 3.5 times more likely to become negative. The current analysis supports these conclusions, while taking into account the randomization and using all locations in the trial.

### Conclusion

This re-analysis of a cluster-randomized trial estimates intervention effects while taking account of spatial spillover, and provides further evidence that deployment of insecticide-treated materials may impact neighbouring locations.

## Supporting information

S1 Text Detailed statistical methods. (PDF)

S1 Data Georeferenced numbers of positive containers per location. A tab-delimited file with three columns: ‘EASTING’, ‘NORTHING’, and finally ‘RESPONSE’ which is the number of containers positive for *Aedes aegypti* immature stages (larvae or pupae) at each location. As explained in the Methods section, some houses had identical GPS coordinates and were merged to the same location.
(TXT)

S2 Data Dirichlet tessellation of the study area, shapefile shape format (.shp). First of a set of four files which make up the ESRI (Environmental Systems Research Institute) shapefile format. To read into R, the four files should be placed in the same folder (directory), for example the working directory, and imported using the ‘readOGR’ function of the ‘rgdal’ package.
(SHP)

S3 Data Dirichlet tessellation, shapefile shape index format (.shx). Second of a set of four files which make up the ESRI shapefile format.
(SHX)

S4 Data Dirichlet tessellation, shapefile attributes format (.dbf). Third of a set of four files which make up the ESRI shapefile format. This file is included for compatibility and does not contain any real attributes of the data.
(DBF)

S5 Data Dirichlet tessellation, shapefile projection metadata format (.prj). Fourth of a set of four files which make up the ESRI shapefile format.
(PRJ)
