# Supplementary material for: Spatial spillover analysis of a cluster-randomized trial against dengue vectors in Trujillo, Venezuela
Source: PLoS Negl Trop Dis. 2020 Sep 3;14(9):e0008576. doi: 10.1371/journal.pntd.0008576 (PMC7494074; doi:10.1371/journal.pntd.0008576)
Supplement: S1 Text — (PDF) [file pntd.0008576.s001.pdf]

Supporting Information S1 Text for Alexander et al.

Model equations

Here we present the main features of the model which is described in more detail in a companion paper [1]. Suppose the trial has n individuals, with count outcomes denoted by $Y_1, Y_2, \ldots, Y_n$ and the corresponding denominators by $L_1, \ldots, L_n$. In the current trial 'individuals' are locations, at which there is usually just one house ($L_i = 1$), although more in the case of identical GPS coordinates. Let $\boldsymbol{c}^T = (c_1, \ldots, c_n)$ be the vector of cluster random effects where $c_i = c_j$ if individuals i and j are both in the same cluster so there are q distinct random effects, one for each cluster ($\boldsymbol{c}^T$ denotes the transpose of $\boldsymbol{c}$) and $\boldsymbol{s}^T = (s_1, s_2, \ldots, s_n)$ a vector of spatially correlated random effects, included to model spillover dependence. Conditionally on all the random effects, $Y_1, \ldots, Y_n$ are assumed independent with Poisson distribution and means given by:

$$E[Y_i \mid \boldsymbol{c}, \boldsymbol{s}] = L_i \exp\left(\alpha + \beta\, t_i + \eta\, d_i\, t_i + \gamma\, d_i\, (1 - t_i) + c_i + s_i\right) \tag{1}$$

Hence the logarithmic link is used. The binary variable t_i indicates whether individual i is in the intervention ($t_i = 1$) or the control arm ($t_i = 0$), and d_i is the number of individuals within a specified radius who are in the intervention arm

We use $N_m(\boldsymbol{0}, \boldsymbol{Q})$ to denote the multivariate normal distribution of dimension m with zero mean vector and covariance matrix $\boldsymbol{Q}$, and $\boldsymbol{I}_m$ denotes the identity matrix of dimension m. We restrict the cluster and spatial random effects to have linear form, namely

$$\boldsymbol{c} = \boldsymbol{Z}_c\, \boldsymbol{a}\,, \qquad \boldsymbol{s} = \boldsymbol{Z}_s\, \boldsymbol{b} \tag{2}$$

where $\boldsymbol{Z}_c$ and $\boldsymbol{Z}_s$ are design matrices with q and k explanatory variables respectively. k is a number that is determined by a) orthogonality constraints and b) restricting the analysis to positive spatial dependence.

The vectors $\boldsymbol{a}$ and $\boldsymbol{b}$ have independent multivariate normal distributions with mean zero, and covariance matrices $\sigma_c^2\, \boldsymbol{I}_q$ and $\sigma_s^2\, \boldsymbol{I}_k$ respectively (where $\boldsymbol{I}_q$ is the $q \times q$ identity matrix). The cluster design matrix $\boldsymbol{Z}_c$ contains q cluster-level covariates where the lth covariate is a vector with 1s for individuals in cluster l, and 0s elsewhere

The spatial effects design matrix $\boldsymbol{Z}_s$ is restricted to give ICAR spatial dependence, and also to satisfy $\boldsymbol{X}^T \boldsymbol{Z}_s = \boldsymbol{0}$ so its columns are orthogonal to those of $\boldsymbol{X}$ where $\boldsymbol{X}$ is the design matrix of fixed effects. The orthogonality between fixed and random effects design matrices is required to avoid so-called spatial confounding [2].

Prior specifications

One of the main requirements for an efficient application of INLA is that the number of hyper parameters be small $-$ less than six in practice. We have three: σ_c^2 and σ_s^2, with the third representing variation between pairs of clusters.

In our novel model, which in the companion paper we call the extended model [1], we assign a flat improper prior for α and independent $N_1(0, 0.001)$ priors for β, η and γ. For the three random effect standard deviations we assign independent exponential priors [3]. This allows small values of σ_r — preventing the priors from imposing extra-Poisson variation — and avoids large values — reflecting the prior belief that the fixed effects are sufficient to explain the data citeKelsall1999. To maintain this behaviour for four random effect precisions, we multiply their scale value of 2000 by $4^2 = 16$, while keeping 0.5 for the shape parameter. These are called penalised

complexity priors [3], and favour the standard model (with no spatial random effects) in order to avoid fitting artificial spatial and cluster dependence. The companion paper [1] contains results from three sets of priors, corresponding to weak, medium and strong penalties to the spatial model as compared to the standard model. For the current dataset the results are similar, and here we use only the medium set.

Goodness of fit

To check the goodness of fit of the models presented in this paper we use posterior predictive diagnostics as opposed to the *deviance information criterion* (DIC) which is known for underpenalizing complex models with many random effects [5]. Here we focus on the *conditional predictive ordinates* (CPOs) which are the leave-one-out cross validation posterior predictive distributions. Each CPO is the posterior probability of obtaining the value of Y_i when the model is fitted to all data except Y_i. A larger value implies a better fit of the model, and very low CPO values suggest that Y_i is an outlier or an influential observation. These quantities can be computed efficiently using INLA [6]. We use the geometric mean of the CPOs as a global measure of goodness of fit. This measure lies in $(0, 1)$ and equals the exponential of the *average log pseudo marginal likelihood* or LPML [7].

References

1. Anaya-Izquierdo K, Lenhart A, Alexander N. Spatial regression and spillover effects in cluster randomised trials with count outcomes. Biometrics. In press.
2. Hodges JS, Reich, B.J. Adding spatially-correlated errors can mess up the fixed effect you love. The American Statistician. 2010;64(4): 325–334.
3. Simpson D, Rue H, Riebler A, Martins TG, Sørbye SH. Penalising model component complexity: A principled, practical approach to constructing priors. Statistical Science. 2017;32:1–28.
4. Lindgren, F. Continuous domain spatial models in R-INLA. ISBA Bulletin. 2012;19(4).
5. Plummer, M. Penalized loss functions for Bayesian model comparison. Biostatistics. 2008;9(3): 523–539.
6. Held L, Schrödle B, Rue H. Posterior and Cross-validatory Predictive Checks: A Comparison of MCMC and INLA. In: Kneib T, Tutz G, editors. Statistical Modelling and Regression Structures. Physica-Verlag HD; 2010. p. 91–110.
7. Geisser S, Eddy W. A predictive approach to model selection. Journal of the American Statistical Association. 1979;74:153–160.
